# Reviews and Book Notices

**Published:** 1882-02

**Authors:** 


### Reviews and Book Notices.

ARTICLE XIV.—AMERICAN NERVOUSNESS. By G. M. Beard, A.M., M.D. 12mo. New York: G. P. Putnam's Sons. 1881.

Before any one can conceive of matter as center of forces, intersections of forces, or condensations of modes of force, he must first take a view of several of the elementary substances, look at them as separate entities, and see in what they resemble one another. He has to do the same with all modes of force before understanding the co-relation of forces. Before one is capable of understanding how species have been evolved by natural selection, he must acquire a few general notions regarding various species of organisms; and before a physician is prepared to investigate neurasthenia, he must be acquainted with nervous diseases in general. And so with *evolution* in all things. It is simply a deeper study, an insight into points of little bearing on the course of nature, since their results become sensible only after eons, though perpetually at work. Hence it is no wonder if we daily meet men of very limited knowledge, who tell us that they have attained the maximum point in wisdom; they are evolutionists; and they wonder how it happened that so many have borrowed their ideas. Yet, on practical questions, these men are found lacking in data, investigation or even memory. They only know the bearings of evolution.

Dr. Beard, in assuming the creation of nervousness in America, and relating its epidemic spread over Europe since, and in alluding to the legions of writers who have borrowed his ideas, lays himself open to as severe criticism as he has met. But evolutionists are so fashionable to-day, that naturally his books on nervousness—small, cheap, and devoid of technicalities—should enjoy many new editions. They are at least a higher grade of pamphlets than the average advertising media of those specialists who treat *manhood lost*, although addressed to the public, as are the latter.

We will review this work in a spirit of evolution, down from evolution of matter to the evolution of insanity, and up to the evolution of the immortal soul, if necessary; but, granting that the leading principles of the book may be correct, we will mention all the deficient links, if possible, which should become a philosophical work, even when addressed to the American public.

This is a *supplement* to a *smaller* work, which we had the pleasure of reviewing a few months ago. But it is also the etiology of the disease considered in the other volume. This is retrogressive evolution, at least, if not disintegration in bookmaking. This new volume is also of a more philosophical (?) and popular character than the other. Follows an epitome of said philosophy.

1. Nervousness, the author says, is strictly deficiency or lack of nerve-force. It would not be difficult to show that the reverse is at least as true, and partly by quoting from his works; but every one knows that nervousness is more generally a superabundance of nerve-force misapplied. Thus, the nervous and mental diseases are more developed in those animals whose nerve-force is the greatest, as we see in Lindsay's Mind in the Lower Animals, Vol. II. Nervousness is also most developed, according to Dr. Beard himself, in the higher races, in which the nerve-force is also the greatest. We might also derive proofs from comparative anatomy to show that the author's proposition is very deficient, and that so must be the idea which underlies it, unless the fault is in the mode of expression; and one is inclined to think that such is the case.

2. The chief and primary cause of this is *modern civilization*. This is another paradox. If there is any stimulant and promotor of nerve-force, it is certainly the highest civilization. This the author would probably confirm, and own that his arbitrary division of said civilization into the five characteristics—steam-power, press, telegraph, the sciences, and the mental activity of woman—is at least very heterogeneous and deficient; and many factors, such as religion, morality, continence, modes of education, and inheritance, have a greater bearing on one's nervousness than the steam-engine, although regarded as secondary and tertiary factors by the author.

3. Indeed, these are harmless, if not interwoven with the modern form of civilization (?).

4. Neurasthenia, evolved of nerve sensitiveness, is the natural basis whence certain physical forms of hysteria, hay-fever, sick headache, inebriety and some phases of insanity evolve. This is an important fact to ascertain.

5. "The greater prevalence of nervousness in America is a complex resultant of a number of influences, the chief of which are dryness of the air (?), extremes of heat and cold, civil and religious liberty, and the great mental activity made necessary and possible in a new and productive country under such climatic conditions." There is much truth in this. "Nervousness, in its extreme manifestations, seems to save one from ataxia, spinal meningitis, and interior spinal sclerosis." "Nervousness of constitution is, indeed, an aid to longevity, and in various ways; it compels caution, makes imperative the avoidance of evil habits, and early warns us of the approach of peril. Probably no great class of people in the world live longer than the professional and business men of America—the very class among whom these nervous disorders are so often found, the class that supplies the victims for our inebriate asylums." "Chewing is very rapidly going out of custom, and will soon, like snuff-taking, become a historic curiosity." Drinking is also on the eve of disappearance. Instead of referring such facts to an increase of nerve-force, far-sightedness, and progress in life and morality, caused by education and will-power, the author refers them to his favorite disease.

Americans, of the present generation, need only small doses of cathartics; and a professional man was unable to bear iron; it made his headache; nor quinine, it made him crazy; nor zinc, it irritated his stomach. "No people in the world drink so little fluid as we. In America pork, like the Indian, flees before civilization."

Constitutional diseases prevent local diseases and *vice versa*. This is a very doubtful proposition.

Early and rapid decay of the teeth; baldness; sensitiveness to heat and cold; thanks to neurasthenia, the Americans are comparatively free from gout and rheumatism.

The eulogy of American beauty by the author is charming, and founded on facts; it almost makes his work one of æsthetics. Dr. Beard assures us that the humorists are in greater demand as lecturers than philosophers, men of science, or of literature; however, we are of the opinion that a popular audience is still better entertained by a circus; but this is not confined to America.

Syphilis is growing milder because of the increasing nervousness of our time. It is comforting news for some to learn that brain-workers enjoy long life, and the chapter treating of this fact is one of the most interesting of the whole book.

In fact, the title, American Nervousness, is ill chosen, unless it means a perspective of the various changes taking place in the higher races, seen through "American Nervousness." As popular reading, although not humorous, this book is very interesting; but it lacks the philosophy of which it boasts. We anticipated that the author would take into consideration the biological fact that many nervous troubles make their appearance in families which are becoming extinct, as some diseases appear in species which are disappearing; that, on the other hand, with a diminution in the number of the offspring, there must be increase of vitality, characterized by longevity, beauty, talents, etc.; and one will question, perhaps with the author, whether neurasthenia is not a quality, a promoter of vitality, rather than a disease. A potent factor, not enough insisted upon, is the fact that a *complete* American individual consists of a few generations, not of one alone; that is, a first generation accumulates money, the next acquire learning, and the third is Dr. Beard's neurasthenic, while on the whole it is certainly the best representative of the highest civilization. H. D. V.

ARTICLE XV.—ARTIFICIAL ANÆSTHESIA AND ANÆSTHETICS. By HENRY M. LYMAN, A.M., M.D., Professor of Physiology and of Nervous Diseases, Rush Medical College; also, Professor of Theory and Practice, Woman's Medical College, Chicago, Ill. New York: Wm. Wood & Co. 1881.

Prof. Lyman has had a task of no small proportions, to group together in a systematic manner the excellences of the different authorities on anæsthesia. That he has performed this task well is apparent to any one who carefully examines this volume.

He has been successful not only as a compiler, but also as a patient, careful and original investigator. He gives the results of a series of experiments on animals and the human subject with chloroform, ethylic bromide, alcohol, ether, amylic nitrite and nitrous oxide. Sphygmographic tracings were taken during the different stages of anæsthesia, which are interesting, if not instructive.

After giving the history and phenomena of anæsthesia, the author discusses the physiology of anæsthesia. This is one of the most interesting chapters in the book. His physiological explanations in regard to sleep and anæsthesia are especially good. He argues that anæsthetic substances diminish or retard the specific molecular movements of nervous or muscular tissues. He says: "When it is remembered that the effect of an anæsthetic is temporary, and that it leaves the tissues in no way different from their original condition, it seems more probable that in the substance of the protoplasm through which the paralyzing agent is diffused, the anæsthetic operates to arrest those chemical changes—chiefly oxidation—that are associated with the normal diffusion of motion throughout the system. It effects no new combinations or decompositions among the molecules; it acts merely the part of a cloud between the sun and the earth, hindering the energies of the one from acting upon the susceptible matter of the other."

This chapter is followed by those on the administration of anæsthetics, inhalers, accidentsof anæsthesia and their treatment, anæsthetic mixtures, anæsthetics in obstetric and dental practice, local anæsthesia, mortality of artificial anæsthesia, medico-legal relations of anæsthesia, anæsthesia by rapid respiration and electricity, and a list of forty-seven anæsthetic substances, with their properties.

The author's style is so clear and even that it it is a pleasure to follow him in his most abstruse reasoning.

J. A. R.

ARTICLE XVI.—A TREATISE ON THE DISEASES OF INFANCY AND CHILDHOOD. By J. L. Smith, M.D. Fifth edition, thoroughly revised, with illustrations.

This is one of the leading text-books on the subject, and is highly recommended by professors to students. Unless one desires to make a specialty of that branch of medicine, it is also all that a general practitioner needs for reference. It is a good supplement to any work on Practice, none of which would be complete without it, or some analogous work. It must not be forgotten that more than one-third of patients are children, and although they must be treated "on general principles," these are not at all sufficient to answer the special circumstances of childhood which give to all diseases very different features from what they have in the adult, not to mention a variety of diseases which are peculiar to childhood alone. The illustrations are only thirty in number, and might as well have been omitted, for, as a rule, they do not elucidate the text of works on Practice, and are not expected to adorn them.

This edition is printed in a smaller type than the previous one, and "leaded," and presents a very good appearance. Indeed, this is a very handsome volume of 836 pages, bound in half Russia, with stained edges, in accordance with a new style of binding, started a few months ago by the publishers: Henry C. Lea's Son & Co., Philadelphia.

ARTICLE XVII.—A SYSTEM OF SURGERY, THEORETICAL AND PRACTICAL. In Treatises by various Authors. Edited by T. Holmes, M.A. First American, from Second English Edition, by John H. Packard, A.M., M.D., assisted by a large corps of the most eminent American Surgeons. In three volumes, with many illustrations. Vol. I.—General Pathology. Morbid Processes. Injuries in General. Complications of Injuries. Injuries of Regions. 8vo, pp. 1008, two-columned, half Russia. Philadelphia: Henry C. Lea's Son & Co. 1881.

The case of the late lamented President Garfield has proved that there is a demand for a higher knowledge of surgery, and also of diseases of the mind, in the United States. Should our knowledge of surgery be considered inferior to that of our English or French colleagues (a conclusion which may not be justifiable), it might be accounted for by the fact that medicine and surgery here have not yet evolved into that higher sphere when one's brain would be insufficient to pursue the study of both.

The list of English contributors contains such names as those of Burdon-Sanderson, Sir James Paget, T. Holmes, C. H. Moore, De Morgan, and eighteen other experienced surgeons, while the American revisers to this volume are such well-known writers as J. H. C. Sims, W. Hunt, James Nevins Hyde, M. Longstreth, S. Ashhurst, L. A. Stimson, J. H. Packard, J. S. Jewell, R. Bartholow, and a few other celebrities.

This work is cyclopædic in character, and every subject is treated in an exhaustive manner. It is specially designed for a reference book which every practicing surgeon should have under hand in cases which require more than ordinary knowledge. The illustrations consist of nine chromo-lithographic plates of great beauty, and 240 wood cuts.

Inflammation, Syphilis, Tumors, Wounds, Fractures, Dislocations and Injuries are all treated in this volume, besides other important matter; and an index is appropriately added at the end of the volume. H. D. V.

ARTICLE XVIII.—A TEXT-BOOK OF PRACTICAL HISTOLOGY, WITH OUTLINE PLATES. By W. Stirling, M.D., S.C.D., With thirty outline plates, one colored plate and twenty-seven wood engravings. Quarto, cloth, pp. 130; $4.50. Philadelphia: J. B. Lippincott & Co. 1881. Chicago: Jansen, McClurg & Co.

This is a book for students, and the outline plates are all intended to be filled by him—a manner of studying which has never been received with favor in the cramming medical colleges now so common. Yet this is certainly the surest means to acquire a practical and thorough knowledge of histology. Such a work is invaluable for those practitioners who delight in the use of the microscope; and the clear type, comprehensive arrangement and accurate delineations lend a charm to a study otherwise dry and uninteresting. The introduction of this book in medical colleges is very desirable, and should be specially encouraged by professors of histology.

Article XIX.—Introduction to Pathology and Morbid Anatomy. By T. H. Green, m.d. Fourth American, from the fifth and enlarged English edition. With 138 fine engravings. 8vo, cloth, pp. 348, $2.25. Philadelphia: Henry C. Lea's Son & Co. 1881. Chicago: Jansen, McClurg & Co.

An old professor of the practice of medicine used to teach treatment in these few words: "Gentlemen, take your pathology and treat the case accordingly." If his students had all possessed the book under consideration it might have easily supplemented the ignorance of the lecturer in matters of treatment; for this is no common work, no hypothetical treatise, but embodies a wonderful amount of knowledge in so small a compass, and treated in such a clear style that every physician should supply himself with every new edition as it comes out.

No late discovery or investigation has been overlooked, and the illustrations are the best that ever accompanied any book on the subject. In few departments of medicine has so much progress been made of late as in that of pathology, supported as it is by the promising sciences of biolology and physiological chemistry, and these lie at the basis of scientific treatment. It will repay most practitioners to peruse this book at least two or three times.

Article XX.—The Microscope and Its Revelations. By W. B. Carpenter, c.b., m.d., ll.d., etc. Sixth edition. Illustrated by twenty-six plates and 500 wood engravings. 12mo, cloth, pp. 882. Philadelphia: Presley Blakiston. 1881. Small type.

A passing notice of this voluminous work is all that should be expected. It is acknowledged to be the most comprehensive work on the subject in the English language. The reputation of the author is world-wide, and through his profound knowledge of physiology this work has been made to suit the medical more than any other scientific profession. It is also indispensable to the biologist, and its researches reach far into palæontology. The reader ought to remember, however, that some assertions met with in this work are based on unascertained facts, and some are opposed to the conclusions of men who, in their special studies, have penetrated deeper than the author. H. D. V.

